# AgdbNet – antigen sequence database software for bacterial typing

**DOI:** 10.1186/1471-2105-7-314

**Published:** 2006-06-21

**Authors:** Keith A Jolley, Martin CJ Maiden

**Affiliations:** 1 The Peter Medawar Building for Pathogen Research and Department of Zoology, University of Oxford, South Parks Road, Oxford, OX1 3SY, UK

## Abstract

**Background:**

Bacterial typing schemes based on the sequences of genes encoding surface antigens require databases that provide a uniform, curated, and widely accepted nomenclature of the variants identified. Due to the differences in typing schemes, imposed by the diversity of genes targeted, creating these databases has typically required the writing of one-off code to link the database to a web interface. Here we describe agdbNet, widely applicable web database software that facilitates simultaneous BLAST querying of multiple loci using either nucleotide or peptide sequences.

**Results:**

Databases are described by XML files that are parsed by a Perl CGI script. Each database can have any number of loci, which may be defined by nucleotide and/or peptide sequences. The software is currently in use on at least five public databases for the typing of *Neisseria meningitidis*, *Campylobacter jejuni *and *Streptococcus equi *and can be set up to query internal isolate tables or suitably-configured external isolate databases, such as those used for multilocus sequence typing. The style of the resulting website can be fully configured by modifying stylesheets and through the use of customised header and footer files that surround the output of the script.

**Conclusion:**

The software provides a rapid means of setting up customised Internet antigen sequence databases. The flexible configuration options enable typing schemes with differing requirements to be accommodated.

## Background

The wide availability of molecular techniques, especially high-throughput nucleotide sequence determination, has enabled various typing schemes that were initially based on the reaction of bacterial surface proteins with immunological reagents to be redefined on the basis of the deduced peptide sequences of the variants targeted. This paradigm shift has generated a need to make variant sequences publicly available to facilitate the identification of known variants and ensure the integrity of a unified nomenclature system. Web-accessible databases that archive nucleotide or peptide sequence data are an ideal means of achieving this. A challenge for the design of generic software for such databases is presented by the fact that schemes vary in the way that variants are defined and in the number of loci that may make up a 'strain' definition. For example, some schemes involve the identification of short peptides located in one or more surface-exposed loops of an antigen [1,2]; whereas others may use larger nucleotide sequences [3] or indeed peptide sequences often in conjunction with corresponding nucleotide sequences [4,5].

For all typing schemes, it is essential that there is broad acceptance on the definition of variants and a central repository of variant designations needs to be maintained and curated for accuracy. This is preferable to the deposition of a variant sequence in an archival database such as Genbank, where no checks are made on sequence quality and the submitter is free to define a variant as they may wish. Because of the variation in schemes, setting up specialised databases usually requires bespoke code to be written for the interfaces between the web server and database engine. Here we describe a configurable software package that enables the rapid construction of these types of sequence databases, allowing queries with either nucleotide or peptide sequences, multiple loci to be queried together and the sequences to be made available for download.

## Implementation

The agdbNet package runs on Linux or UNIX systems using the PostgreSQL database and Apache web server. The core software is written in Perl as a single, mod_perl compatible, CGI web script that interfaces with BLAST [6]. BLAST is an essential component of the system, but other applications may be optionally installed to enhance functionality; for example, EMBOSS [7] is used to generate sequence alignments of nearest alleles and peptides, and Bioperl [8] allows sequences to be downloaded in multiple formats. A configuration file defines the paths for BLAST and the other helper applications, working directories and site-wide options.

The software uses XML configuration files to describe the structures of individual databases. The XML parsing functionality was derived from code written for use with multilocus sequence typing databases [9,10]. Every database XML file has a <system> tag that contains database-specific configuration options such as the name of the database, the local path to the web root and a text description of the database. There will also be at least one set of <locus> tags, enclosing either <peptide> or <nucleotide> tags (or both) that describe sequence tables. Any number of fields may be defined within these tables, and options set for whether they are displayed in the main results table following a query. Databases can also contain an isolate table containing information about representative or reference isolates that exhibit a given antigen. It is also possible to define an external isolate database table that can be queried for a matching antigen. Database searches on external systems require the remote system to be configured to allow connections on the PostgreSQL port and remote queries to the particular database in question.

In order to add to and edit the database, a Perl script is provided to run a private web interface for the curator. The interface enables sequences to be added rapidly and automatically performs a data integrity check. The curator's interface script reads the same XML file as the main website script, so that any modifications are kept in sync. The curator can run an arbitrary script on the system by activating a button on the curator's interface, if the script's path has been defined in the XML file. This script enables the updating of static web pages from the database, for instance, without requiring the curator to have administrator access to the system.

The software produces standards-compliant XHTML and uses cascading style sheets (CSS) so that the style of the resulting website can be modified easily. Additionally, header and footer HTML files can be defined that will be added to the resulting pages so that they can conform to the layout of a particular website, enabling the look-and-feel to be modified easily.

## Results and discussion

### Public databases using this software

**Figure 1:**
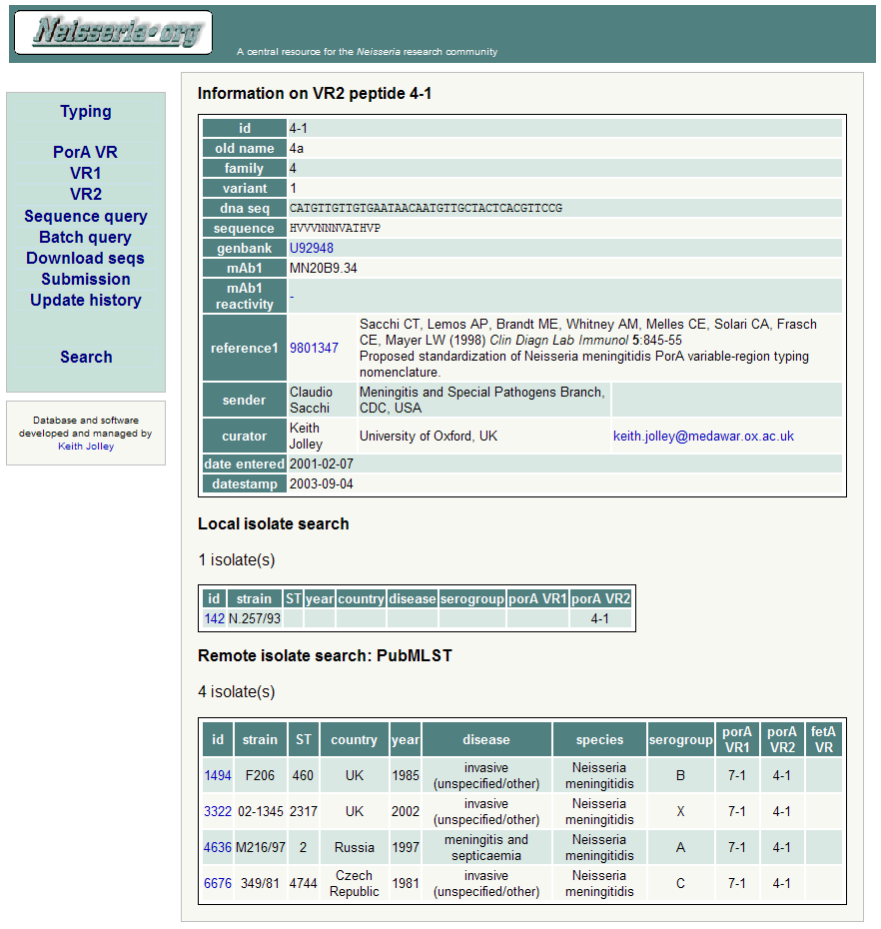
**Screenshot: *Neisseria meningitidis *PorA variable region database**. Results following a hyperlink for a particular variant sequence. The resulting page lists all known information about the variant, such as who first reported it, where it has been published, accession numbers and its monoclonal antibody reactivity. Isolates stored within the PorA database that express the variant are shown and then the results of a similar search against the external PubMLST isolate database.

The software is in use on a number of public bacterial typing databases. The first site to be implemented was the PorA variable region database for subtyping *Neisseria meningitidis *[1,11], a major cause of meningitis and septicaemia. The PorA protein is a major typing target and vaccine candidate. This scheme defines the peptide variants at two variable regions (VR1 and VR2). Either nucleotide or peptide sequences can be queried against both loci, either singly or, more usually, together. If a variant is identified, a hyperlink will lead to a page describing all the information known about it, including antibody reactivities, Genbank and PubMed accession numbers and links, and the submitter information (figure 1). Along with the peptide information, a table listing known isolates expressing the variant is shown. Further information about the isolates can be displayed by following the hyperlinks from this table. The software will also query the external PubMLST isolate database [10,12] listing isolates from it that also match [see Additional file 1: poravr.xml for the XML description of this database].

Databases for other *Neisseria *antigens are also available [11]: i) a nucleotide database for the two different classes of the typing antigen PorB [3]; ii) A variable region peptide database for a putative vaccine candidate, FetA [2,3].

A database containing both alleles and peptides for the short variable region of the FlaA typing antigen of *Campylobacter*, an organism frequently implicated in cases of food poisoning, is also available [4,13]. Investigating the diversity in the FlaA protein, coupled to broader typing methods, can enhance the discrimination of isolates during outbreak investigations.

**Figure 2:**
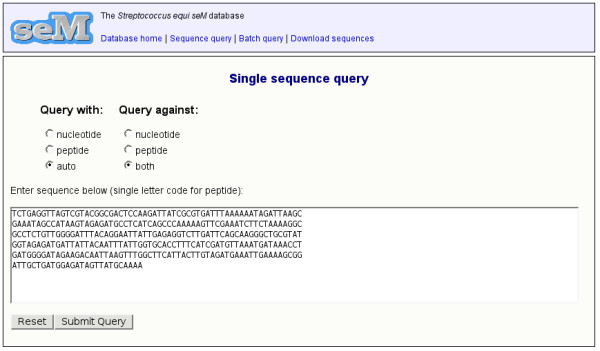
**Screenshot: Querying the *Streptococcus equi *seM database**. A nucleotide sequence has been pasted in to the web form and the selection has been made to query against both the nucleotide and peptide sequence tables.

**Figure 3:**
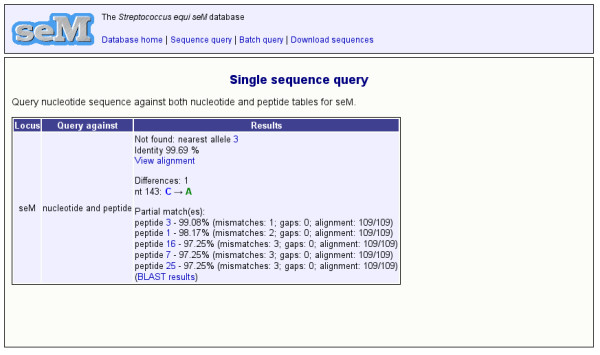
**Screenshot: Results of a search of the *Streptococcus equi seM *database**. The software has identified that the query sequence is not known but is most similar to allele 3. It then shows the nucleotide differences. The BLAST search against the peptide table has produced a number of partial matches. The alignment output from BLAST can be viewed by clicking the appropriate hyperlink. All allele and peptide numbers are hyperlinked to lead to more detailed information about the sequence.

Recently, a database for a sensitive subtyping scheme for *Streptococcus equi*, the causative agent of strangles in horses, has been set up that indexes the variation found in the SeM protein [5,14] (figures 2 and 3). Use of this scheme has been used to investigate potential cases of disease related to administration of live attenuated *S. equi *vaccine.

### Interconnected distributed databases

Because databases hosted using this software share a common platform, it makes it practical to retrieve information from them by other websites, creating a network of interconnected distributed databases. This can be seen in practice on the multilocus sequence typing (MLST) databases for *Neisseria *[10,12]. If an isolate has been genetically subtyped, the MLST database software will automatically query the PorA variable region database and display a hyperlinked peptide that takes the user to a page on the PorA website describing that peptide. This interconnection works both ways as the PorA website can also query pubmlst.org to list isolates that contain a particular subtype. These interconnections between databases can be configured in the software by a single line in the XML description.

## Conclusion

This software enables the rapid construction of web-based antigen databases. These databases can contain multiple sets of nucleotide or peptide sequences, or both, and may be queried using nucleotide or peptide sequences. Multiple loci may be queried simultaneously, an advantage for typing schemes that involve separate variable regions that may be located within a single larger sequence. The software has been successfully deployed in a number of applications which are being used daily by the worldwide public health and research communities.

## Availability and requirements

Project name: AgdbNet

Project home page: 

Operating systems: Linux/UNIX

Programming language: Perl

Other requirements: Apache; PostgreSQL; CGI, DBI, XML::Parser::perlSAX Perl modules; BLAST

License: GNU GPL

Any restrictions to use by non-academics: none

A distribution archive of the software (version 1.0.0) is available with this manuscript [see Additional file 2].

## Authors' contributions

KAJ carried out the programming work and drafted the manuscript. MCJM conceived the software development and participated in defining its specification. Both authors read and approved the final manuscript.

## Supplementary Material

Additional File 1 XML (text) file showing the configuration for the Neisseria PorA VR database. Click here for file

Additional File 2 Distribution archive of the software (version 1.0.0). Click here for file
