# Supplementary material for: AgdbNet – antigen sequence database software for bacterial typing
Source: BMC Bioinformatics. 2006 Jun 21;7:314. doi: 10.1186/1471-2105-7-314 (PMC1543660; doi:10.1186/1471-2105-7-314)
Supplement: Additional File 2 — Distribution archive of the software (version 1.0.0). [file 1471-2105-7-314-S2.gz › agdbnet_v1.0.0/installation.html]

AgdbNet installation


# AgdbNet installation

## Instructions for setting up agdbNet software

1. Copy the distribution package to a temporary directory.
2. Unpack the package using the following commands:  

   gunzip agdbnet.tar.gz  
   tar xvf mlstdbnet.tar
3. Copy the agdbnet.pl script from the cgi-bin directory into a subdirectory of the cgi-bin directory of your
   web server. Make sure the file is readable
   and executable by everyone:

   chmod a+rx agdbnet.pl
4. Copy the contents of the conf directory to /usr/local/agdbnet - this location can be changed by
   modifying the path to agdbnet.conf at the beginning of the agdbnet.pl script. Edit the agdbnet.conf
   file to suit your configuration - set the paths to any helper programs/modules installed or switch off functionality.
5. Create a temporary directory called tmp, allowing read and write access to the web server user (e.g. apache or www-data) within the web root, e.g. /var/www/html/tmp. Set the path to the temporary directory in the agdbnet.conf file.
6. If you want to include PubMed reference fields in your database, create an empty reference database 'refs' by using the refs.sql script (setup directory) from within PostgreSQL.
   To populate the refs database, run the getrefs.pl script as a nightly cron job once
   isolate databases have been set up.
7. Create XML database description file
8. Copy the XML file to the cgi-bin directory containing the agdbnet.pl script.
9. Create the SQL file to generate the database using the xml2sql.pl script found in the scripts directory. First make sure that the XML has a valid syntax by running the script as below:  

   ./xml2sql.pl database.xml

   where 'database.xml' is the XML file you created above (change the filename as appropriate). If the script does not produce an error message, generate your SQL file as follows - substitute the name of the web server user account for 'apache':  

   ./xml2sql.pl -u apache database.xml > database.sql
10. Create the database - you may need to log in with an account with permissions to create new databases, e.g. 'postgres':  

    createdb database

    where 'database' is the name of the database you want to create. Run psql and generate the database by running the SQL script:  

    psql database  
    \i database.sql  
    \q
11. Create an HTML index page for your website. From the index page, link to the script entry page for the database,
    which is located at 'http://webroot/cgi-bin/agdbnet/agdbnet.pl?file=XMLFILE.xml' where XMLFILE.xml is the XML file describing the database.
12. Customise the look of the website. The script looks for a cascading stylesheet called stylesheet.css in the WEBROOT/database/ directory (where 'WEBROOT' is the root of the web site and 'database' is the name of the database you've created). You can also put a header.html and a footer.html file in this directory - the contents of these files will surround the output of the script. Additionally, you can put a banner.html file in this directory to display text on the script index page.
13. To insert and edit data, put the agcurate.pl script in a password-protected cgi-bin directory. Create a symlink to the xml file in this directory. The software uses apache authentication to check that the user has rights to curate the database. You therefore need to add a user to the users table of the database with a matching username to the one that you have logged in with. To do this, log in to the database with psql and directly insert as follows:  

    psql database  
    INSERT INTO users VALUES (1, 'keith', 'Jolley', 'keith', 'keith.jolley@medawar.ox.ac.uk', 'University of Oxford', 'curator', 'today', 'keith');  
    \q

    where the order of fields is id, username, surname, first name, email, affiliation, status, datestamp, curator. All other changes to the database can be made by navigating to the script, e.g. 'http://webroot/cgi-bin/private/agcurate.pl?file=XMLFILE.xml'. The curator's site can also have header and footer files that surround the output of the script - create curate\_header.html and curate\_footer.html files in WEBROOT/database/.
